# Antimicrobial and Insecticidal: Cyclic Lipopeptides and Hydrogen Cyanide Produced by Plant-Beneficial *Pseudomonas* Strains CHA0, CMR12a, and PCL1391 Contribute to Insect Killing

**DOI:** 10.3389/fmicb.2017.00100

**Published:** 2017-02-03

**Authors:** Pascale Flury, Pilar Vesga, Maria Péchy-Tarr, Nora Aellen, Francesca Dennert, Nicolas Hofer, Karent P. Kupferschmied, Peter Kupferschmied, Zane Metla, Zongwang Ma, Sandra Siegfried, Sandra de Weert, Guido Bloemberg, Monica Höfte, Christoph J. Keel, Monika Maurhofer

**Affiliations:** ^1^Plant Pathology, Institute of Integrative Biology, ETH ZürichZürich, Switzerland; ^2^Department of Fundamental Microbiology, University of LausanneLausanne, Switzerland; ^3^Laboratory of Experimental Entomology, Institute of Biology, University of LatviaRiga, Latvia; ^4^Laboratory of Phytopathology, Faculty of Bioscience Engineering, Ghent UniversityGhent, Belgium; ^5^Microbial Biotechnology and Health, Institute of Biology Leiden, Leiden UniversityLeiden, Netherlands

**Keywords:** orfamide, sessilin, Gac regulatory system, *Pseudomonas fluorescens*, *Pseudomonas protegens*, *Pseudomonas chlororaphis*, secondary metabolites, insecticidal activity

## Abstract

Particular groups of plant-beneficial fluorescent pseudomonads are not only root colonizers that provide plant disease suppression, but in addition are able to infect and kill insect larvae. The mechanisms by which the bacteria manage to infest this alternative host, to overcome its immune system, and to ultimately kill the insect are still largely unknown. However, the investigation of the few virulence factors discovered so far, points to a highly multifactorial nature of insecticidal activity. Antimicrobial compounds produced by fluorescent pseudomonads are effective weapons against a vast diversity of organisms such as fungi, oomycetes, nematodes, and protozoa. Here, we investigated whether these compounds also contribute to insecticidal activity. We tested mutants of the highly insecticidal strains *Pseudomonas protegens* CHA0, *Pseudomonas chlororaphis* PCL1391, and *Pseudomonas* sp. CMR12a, defective for individual or multiple antimicrobial compounds, for injectable and oral activity against lepidopteran insect larvae. Moreover, we studied expression of biosynthesis genes for these antimicrobial compounds for the first time in insects. Our survey revealed that hydrogen cyanide and different types of cyclic lipopeptides contribute to insecticidal activity. Hydrogen cyanide was essential to full virulence of CHA0 and PCL1391 directly injected into the hemolymph. The cyclic lipopeptide orfamide produced by CHA0 and CMR12a was mainly important in oral infections. Mutants of CMR12a and PCL1391 impaired in the production of the cyclic lipopeptides sessilin and clp1391, respectively, showed reduced virulence in injection and feeding experiments. Although virulence of mutants lacking one or several of the other antimicrobial compounds, i.e., 2,4-diacetylphloroglucinol, phenazines, pyrrolnitrin, or pyoluteorin, was not reduced, these metabolites might still play a role in an insect background since all investigated biosynthetic genes for antimicrobial compounds of strain CHA0 were expressed at some point during insect infection. In summary, our study identified new factors contributing to insecticidal activity and extends the diverse functions of antimicrobial compounds produced by fluorescent pseudomonads from the plant environment to the insect host.

## Introduction

Root-colonizing fluorescent pseudomonads are well known for their plant-beneficial traits, which include inhibition of root-pathogens, induction of resistance in the plant and solubilization of mineral nutrients. Nevertheless, during the last decade evidence arose that we should widen our view on their life-style since plant roots apparently are not the only environment colonized by these bacteria. Indeed, many strains throughout the *Pseudomonas fluorescens* group were discovered to have the ability to colonize insects and strains of two sub-clades can even cause lethal infections (Péchy-Tarr et al., 2008; Olcott et al., 2010; Ruffner et al., 2013, 2015; Flury et al., 2016; Keel, 2016). Especially strains of sub-clade 1 (Loper et al., 2012), also called the *Pseudomonas chlororaphis* subgroup (Gomila et al., 2015), are highly insecticidal when injected, but also when ingested by insect larvae (Ruffner et al., 2015; Flury et al., 2016). How exactly plant-associated pseudomonads colonize insects and which factors are decisive for fatal infections is still largely unknown.

To date the most intensively studied virulence factor against insects is the Fit toxin, which is similar to Mcf1 of the entomopathogenic bacterium *Photorhabdus luminescens* (Péchy-Tarr et al., 2008, 2013; Kupferschmied et al., 2014; Ruffner et al., 2015). Fit toxin-negative mutants of *Pseudomonas protegens* strains CHA0 and Pf-5 exhibited reduced toxicity when injected into the hemolymph of *Galleria mellonella* or *Manduca sexta* larvae (Péchy-Tarr et al., 2008). Fit mutants of *P. protegens* CHA0 and *P. chlororaphis* PCL1391 further showed reduced virulence when fed to larvae of *Spodoptera littoralis* (Ruffner et al., 2013). However, all Fit mutants retained significant virulence, and in oral infections of *Drosophila melanogaster* by *P. protegens* Pf-5 no role for the Fit toxin could be detected (Loper et al., 2016). This indicates the involvement of additional virulence factors and points to a certain specificity of virulence factors to individual insect species (Keel, 2016).

For oral insecticidal activity a functional GacS/GacA-regulatory system is essential (Ruffner et al., 2013; Loper et al., 2016). Accordingly, a Fit-GacA double mutant was strongly reduced in virulence (Ruffner et al., 2013) in oral infections of *S. littoralis*. Recently, chitinase C was identified as one of the Gac-regulated factors contributing to toxicity of *P. protegens* CHA0 and Pf-5 toward *Plutella xylostella* and *D. melanogaster*, respectively (Flury et al., 2016; Loper et al., 2016). While some factors, such as the Fit toxin or chitinase C, are present throughout all strains of the *P. chlororaphis* subgroup, the observation of strain-specific differences in toxicity further suggested the existence of strain-specific factors (Flury et al., 2016). Rhizoxin seems to be such a factor as it strongly contributes to insecticidal activity of *P. protegens* Pf-5, but is not produced by most other strains belonging to the *P. chlororaphis* subgroup (Loper et al., 2016). Generally, antimicrobial compounds produced by strains of the *P. chlororaphis* subgroup, such as 2,4-diacetylphloroglucinol (Phl), pyrrolnitrin (Prn), pyoluteorin (Plt), hydrogen cyanide (Hcn), phenazines (Phz), and cyclic lipopeptides (Clp) represent possible candidates for a role in insecticidal activity. These compounds exhibit toxic effects toward a broad spectrum of organisms (Gross and Loper, 2009) and their production is Gac-regulated (Hassan et al., 2010; Kidarsa et al., 2013). Besides their well-demonstrated contribution to biocontrol activity of pseudomonads against fungal pathogens, also activity against bacteria, protists, nematodes, arthropods, plants, and mammalian cells is reported (Keel et al., 1992; Maurhofer et al., 1992, 1995; Devi and Kothamasi, 2009; Gross and Loper, 2009; Neidig et al., 2011; Nisr et al., 2011; Jang et al., 2013).

The aim of this study was to investigate whether antimicrobial compounds are also important for these pseudomonads when infecting an insect host. The highly insecticidal *P. chlororaphis* subgroup includes three species: *P. protegens, P. chlororaphis* and a yet to be named species comprising strains such as *Pseudomonas* sp. CMR12a and *Pseudomonas* sp. CMR5c (Flury et al., 2016). Since the different species produce distinct sets of antimicrobial compounds we selected one representative strain per species: *P*. *protegens* CHA0, *P. chlororaphis* PCL1391, and *Pseudomonas* sp. CMR12a. All three strains produce Hcn and *P. protegens* CHA0 additionally produces Phl, Plt, and Prn while *P. chlororaphis* PCL1391 and *Pseudomonas* sp. CMR12 produce phenazine-1-carboxylic acid and phenazine-1-carboxamide (Chin-A-Woeng et al., 1998; Haas and Keel, 2003; Perneel et al., 2007). CMR12a further produces two Clps, orfamide (Ofa) and sessilin (Ses) (D'aes et al., 2011, 2014), the latter of which is closely related to tolaasin of the mushroom pathogen *Pseudomonas tolaasii* (Bassarello et al., 2004). Recently, orfamide production was also demonstrated for *P. protegens* CHA0 (Ma et al., 2016) and *P. chlororaphis* PCL1391 was found to harbor genes for the synthesis of a Clp (Flury et al., 2016). Here, we screened newly generated as well as existing mutants deficient for one or several of the different antimicrobial metabolites for their toxic activity toward insects either when injected directly into the hemocoel or when taken up orally. Furthermore, virulence of mutants deficient for additional compounds or enzymes contributing to biocontrol, i.e., pyoverdine (Pvd), extracellular protease AprA (AprA), glucose dehydrogenase (Gcd), and gluconate dehydrogenase (Gad) (Keel et al., 1989; Siddiqui et al., 2005; De Werra et al., 2009), that potentially could give an advantage during insect colonization, was investigated. Finally, expression of biosynthetic genes for a selection of the investigated metabolites was studied in CHA0 during the process of insect infection.

Our findings highlight major contributions to insecticidal activity for all investigated Clps as well as for Hcn, but not for any of the other metabolites or enzymes.

## Materials and methods

### Bacterial growing conditions

The bacterial strains used in this study are listed in Table 1. For all insect assays bacterial strains taken from our long-term strain storage at −80°C were grown for 2 days at 24°C on King's medium B agar (King et al., 1954) supplemented with ampicillin (40 μg ml^−1^), chloramphenicol (13 μg ml^−1^), and cycloheximide (100 μg ml^−1^) or supplemented with kanamycin (50 μg ml^−1^) depending on the strain. These cultures were used to inoculate 10 mL LB medium (Bertani, 1951). Bacterial cultures were incubated overnight with rotational shaking (180 rpm) at 24°C. Cells were washed twice or three times in sterile 0.9% NaCl solution for feeding or injection experiments, respectively, and diluted to the required concentration.

**Table 1:** **Strains used in this study**.

**Strain**	**Genotype or phenotype**	**Defective in the production of**	**References or comments**
***Pseudomonas protegens***			
CHA0	Wild type, isolated from tobacco roots		Stutz et al., 1986; Jousset et al., 2014
CHA19	Δ*gacS* deletion mutant of CHA0	GacS sensor	Zuber et al., 2003
CHA89	*gacA*::Km^r^ insertion mutant of CHA0	GacA response regulator	Laville et al., 1992
CHA400	*pvd-400*::Tn*1733* insertion mutant of CHA0; Km^r^	Pyoverdine (Pvd)	Keel et al., 1989
CHA805	Nonpolar *aprA*::'*lacZ* insertion mutant of CHA0	Protease AprA (AprA)	Siddiqui et al., 2005
CHA805-g	CHA805::attTn*7*-*gfp2*, Gm^r^	AprA	This study
CHA1151	Δ*fitD* in-frame deletion mutant of CHA0	Fit toxin (Fit)	Péchy-Tarr et al., 2008
CHA1196	Δ*gcd* in-frame deletion mutant of CHA0	Glucose dehydrogenase (Gcd)	De Werra et al., 2009
CHA1197	Δ*gad* in-frame deletion mutant of CHA0	Gluconate dehydrogenase (Gad)	De Werra et al., 2009
CHA1241	Δ*phlACBD* deletion mutant of CHA0	2,4-Diacetylphloroglucinol (Phl)	This study
CHA1281	Δ*fitD gacA*::Km^r^ mutant of CHA0	Fit toxin and GacA	Ruffner et al., 2013
CHA5091	Δ*prnABCD* deletion mutant of CHA0	Pyrrolnitrin (Prn)	This study
CHA5092	Δ*pltABCDEFG* deletion mutant of CHA0	Pyoluteorin (Plt)	This study
CHA5098	Δ*phlACBD* Δ*prnABCD* Δ*pltABCDEFG* deletion mutant of CHA0	Phl/Prn/Plt	This study
CHA5101	Δ*ofaABC* deletion mutant of CHA0	Orfamide (Ofa)	This study
CHA5103	Δ*hcnABC* deletion mutant of CHA0	Hydrogen cyanide (Hcn)	This study
CHA5118	Δ*phlACBD* Δ*prnABCD* Δ*pltABCDEFG* - Δ*hcnABC* Δ*ofaABC* deletion mutant of CHA0	Phl/Prn/Plt/Hcn/Ofa	This study
***Pseudomonas chlororaphis***			
PCL1391	Wild type, isolated from tomato roots		Chin-A-Woeng et al., 1998
PCL1113	*phzF*:: Tn*5*-*luxAB* insertion mutant of PCL1391; Km^r^	Phenazine-1-carboxylic acid and phenazine-1-carboxamide (Phz)	Chin-A-Woeng et al., 1998
PCL1123	*gacS*::Tn*5-luxAB* insertion mutant of PCL1391; Km^r^	GacS	This study
PCL1832	*PCL1391_3318*::Tn5-luxAB insertion mutant of PCL1391; Km^r^	Clp1391	This study
PCL5103	Δ*hcnABC* deletion mutant of PCL1391	Hcn	This study
***Pseudomonas*** **sp**.			
CMR12a	Wild type, isolated from cocoyam roots		Perneel et al., 2007
CMR12a-ΔPhz	Δ*phzABCDEFGH* deletion mutant of CMR12a	Phenazine (Phz)	D'aes et al., 2011
CMR12a-Clp1	*sesA* insertion mutant, Gm^r^	Sessilin (Ses)	D'aes et al., 2011
CMR12a-ΔPhz-Clp1	Δ*phzABCDEFGH sesA* mutant of CMR12, Gm^r^	Phz/Ses	D'aes et al., 2011
CMR12a-ΔClp2	Δ*ofaBC* deletion mutant of CMR12a	Orfamide (Ofa)	D'aes et al., 2014
CMR12a-ΔPhz-ΔClp2	Δ*phzABCDEFGH* Δ*ofaBC* mutant of CMR12a	Phz/Ofa	D'aes et al., 2014
CMR12a-ΔClp2-Clp1	Δ*ofaBC sesA* mutant of CMR12a, Gm^r^	Ofa/Ses	D'aes et al., 2014
CMR12a-ΔPhz-ΔClp2-Clp1	Δ*phzABCDEFGH* Δ*ofaBC sesA* mutant of CMR12a, Gm^r^	Phz/Ofa/Ses	D'aes et al., 2014
***Escherichia coli***			
DH5α	*recA1 endA1 hsdR17 deoR thi-1 supE44 gyrA96 relA1* Δ(*lacZYA*-*argF*)U169(Φ80d*lacZ*ΔM15)		Sambrook and Russel, 2001
S17-1/λpir	*pro thi hsdR recA chromosome*::RP4-2; Tc::Mu Km::Tn*7*/ λpir; Tp^r^ Sm^r^		Simon et al., 1983

*Gm^r^, gentamicin; and Km^r^, kanamycin resistance, respectively*.

### Construction of deletion mutants of *P. protegens* CHA0 and *P. chlororaphis* PCL1391

Mutants of *P. protegens* CHA0 and *P. chlororaphis* PCL1391 with deletions in the biosynthetic genes for antimicrobial compounds were created by means of an allelic replacement technique using the I-SceI system with the suicide vector pEMG (Martinez-Garcia and De Lorenzo, 2011) as described by Kupferschmied et al. (2014). Briefly, using the primer pairs listed in Supplementary Table S1, the 600–700-bp upstream and downstream regions flanking the genomic region to be deleted were amplified by PCR. After digestion with the indicated restriction enzymes (Supplementary Table S1), the fragments were cloned into pEMG via triple ligation yielding the final suicide plasmid which was verified by sequencing. Next, using the I-SceI system with the expression plasmid pSW-2, the deletion mutants CHA1241, CHA5091, CHA5092, CHA5101, CHA5103, CHA5098, CHA5118, and PCL5103 were generated (Table 1).

### Transposon mutagenesis of PCL1391

Additional mutants of *P. chlororaphis* PCL1391 were generated by random transposon mutagenesis. Plasmid pRL1063A (Wolk et al., 1991), which harbors the Tn5luxAB transposon and a kanamycin marker gene, was used to generate transconjugants of PCL1391. Nalidixic acid was added to a final concentration of 15 μg/mL to reduce growth of the *E. coli* helper strains, carrying pRL1063A and pRK2013 (Ditta et al., 1980). A library of Tn5luxAB mutants of PCL1391 was screened for loss of drop collapsing activity, which resulted in the selection of biosurfactant mutants. To recover the regions flanking the transposon insertion site, chromosomal DNA of the mutant was isolated and digested with EcoRI. After re-circulation and transformation into *E. coli* DH5α, the Tn5luxAB flanking chromosomal DNA regions were sequenced. The primers used, i.e. oMP407 (5′-TACTAGATTCAATGCTATCAATGAG-3′) and oMP408 (5′-AGGAGGTCACATGGAATATCAGAT-3′), were homologous to the left and right border of the Tn5luxAB, respectively. Sequences obtained were analyzed using BLASTX in GENbank (Altschul et al., 1997). Sequence analysis of the Tn5luxAB flanking regions revealed that for one mutant, strain PCL1123, the transposon was inserted at bp 2575/2576 in the global regulatory gene for secondary metabolites, *gacS* (Workentine et al., 2009). For a second mutant (referred to as PCL1832) the transposon was inserted in locus PCL1391_3318 at bp 9621/9622. PCL1391_3318 is homologous (>70% nucleotide sequence identity) to the orfamide biosynthetic gene *ofaC* of *P. protegens* Pf-5 (Gross et al., 2007).

### Hcn production assay

To test for the production of Hcn, bacteria were grown on King's medium B agar. An Hcn-indicator paper (Whatman 3M, soaked in a solution of Cu (II) ethylacetoacetate (5 mg; Kodak) and 4,4′-Methylenebis(*N,N*-dimethylaniline) (5 mg; Fluka) per ml chloroform, dried, and stored in the dark (Castric and Castric, 1983) was placed in the lid of the petri-dish. After incubation at 24°C for 24 h blue coloration indicated Hcn production (Voisard et al., 1989).

### Droplet collapse assay

To test whether bacterial strains were able to decrease the surface tension, the ability to collapse a droplet of water on a Parafilm “M” laboratory film (American National Can, Chicago, IL) was assessed (Jain et al., 1991). Twenty Five or fifty μl King's medium B culture supernatant was pipetted directly onto Parafilm. The flattening and spreading of the droplet (drop collapsing activity) was used as an indicator of biosurfactant production.

### Swarming assay

Surface swarming motility was assessed on soft agar LB plates (0.6% w/v agar). *Pseudomonas* strains were grown in King's medium B with rotational shaking (150 rpm) at 28°C for 24 h. Bacterial cells were washed three times in sterile water and 5 μL of cell suspensions were pipetted onto the center of the plates. For complementation of the Ofa^−^ mutant CHA5101, orfamide A of *P. protegens* CHA0, which was purified as described by Ma et al. (2016), was added to the medium. Pictures were taken after 20 h of incubation at 28°C.

### Galleria injection assay

Injection assays with *G. mellonella* were performed as described by Flury et al. (2016) with small adaptations. Last-instar *G. mellonella* larvae (Hebeisen Fishing, Zürich, Switzerland) were injected with 2 × 10^3^ washed bacterial cells suspended in 10 μl of 0.9% sterile NaCl using a repetitive dispensing Tridak Stepper (Intertronic, Oxfordshire, UK). Injected suspensions were further plated in three dilutions on King's medium B agar to confirm that suspensions of different strains indeed contained the same number of bacteria. Larvae were kept in Petri dishes at 24°C in the dark. When they started to become melanized they were scored as live or dead every hour. Each experiment consisted of three replicates per treatment with 10 larvae per replicate. Every mutant strain was tested at least in three independent experiments and two representative experiments are shown in Table 2. To exclude a human bias, the experiments were performed double-blind, i.e., the set-up of the experiments and the scoring of the larvae was performed without knowledge of the treatments.

**Table 2:** **Lethal time 50 (LT_50_) for *Galleria mellonella* larvae injected with wild-type and mutant *Pseudomonas* strains**.

**Strain**	**Phenotype**	**LT**_**50**_ **(h)**			
		**Experiment 1**	**Experiment 2**	**Experiment 3**	**Experiment 4**
CHA0	Wild type	29.1 (28.8; 29.4)	32.9 (32.5; 33.3)	33.8 (33.4; 34.1)	33.7 (33.3; 34.1)
CHA1151	Fit^−^	30.6 (30.1; 31.1)^*^	32.8 (32.5; 33.2)	34.6 (34.2; 35.0)^*^	36.3 (35.8; 36.7)^*^
CHA1281	GacA^−^Fit^−^	31.8 (31.3; 32.2)^*^	35.3 (34.6; 35.9)^*^	34.1 (33.7; 34.5)	36.8 (36.3; 37.3)^*^
CHA400	Pvd^−^			33.0 (32.4; 33.6)	34.1 (33.6; 34.5)
CHA1196	Gcd^−^			34.7 (34.0; 34.8)	34.7 (34.2; 35.2)^*^
CHA1197	Gad^−^			33.9 (33.4; 34.3)	30.4 (29.8; 30.9)^†^
CHA805-g	AprA^−^			33.6 (33.3; 34.0)	32.7 (32.3; 33.0)^†^
CHA1241	Phl^−^	28.7 (28.3; 29.1)	33.0 (32.6; 33.5)		
CHA5091	Prn^−^	28.5 (28.2; 28.9)	32.7 (32.3; 33.0)		
CHA5092	Plt^−^	28.6 (28.3; 29.0)	30.8 (30.3; 31.3)^†^		
CHA5101	Ofa^−^	28.2 (27.6; 28.8)	30.9 (30.3; 31.4)^†^		
CHA5103	Hcn^−^	30.9 (30.5; 31.2)^*^	35.8 (35.4; 36.2)^*^		
CHA5118	Phl^−^Prn^−^Plt^−^Hcn^−^Ofa^−^	29.3 (28.9; 29.8)	32.6 (32.1; 33.1)		
CHA19	GacS^−^			36.0 (35.3; 36.7)^*^	32.0 (31.5; 32.6)^†^
CHA89	GacA^−^			32.6 (32.2; 33.1)^†^	33.8 (33.3; 34.3)
Control		>40	>40	>38	>38
		**Experiment 1**	**Experiment 2**		
CMR12a	Wild type	26.8 (26.5; 27.0)	29.2 (28.6; 29.8)		
Clp1	Ses^−^	29.4 (29.1; 29.7)^*^	34.3 (33.9; 34.7)^*^		
ΔClp2	Ofa^−^	26.4 (26.2; 26.7)	28.2 (27.8; 28.7)		
ΔClp2-clp1	Ofa^−^ Ses^−^	27.0 (26.7; 27.3)	30.0 (29.7; 30.4)		
ΔPhz	Phz^−^	26.7 (26.5; 27.0)	29.8 (29.5; 30.2)		
ΔPhz-Clp1	Phz^−^Ses^−^	28.5 (28.2; 28.7)^*^	32.0 (31.7; 32.4)^*^		
ΔPhz-ΔClp2	Phz^−^Ofa^−^	26.1 (25.9; 26.3)^†^	29.1 (28.6; 29.6)		
ΔPhz-ΔClp2-Clp1	Phz^−^Ofa^−^Ses^−^	28.8 (28.4; 29.2)^*^	31.9 (31.5; 32.3)^*^		
Control		>39	>39		
		**Experiment 1**	**Experiment 2**	**Experiment 3**	**Experiment 4**
PCL1391	Wild type	28.3 (28.0; 28.6)	29.3 (29.0; 29.7)	28.9 (28.4; 29.4)	26.5 (26.2; 26.8)
PCL1832	Clp1391^−^	30.0 (29.6; 30.4)^*^	32.3 (31.8; 32.7)^*^	31.2 (30.7; 31.4)^*^	28.3 (27.8; 28.7)^*^
PCL1113	Phz^−^	28.8 (28.4; 29.1)	30.0 (29.6; 30.5)		
PCL1123	GacS^−^	29.2 (28.9; 29.5)^*^	30.5 (30.0; 31.0)^*^		
PCL5103	Hcn^−^			32.4 (31.8; 33.0)^*^	27.4 (27.1; 27.8)^*^
Control		>39	>39	>33.5	>34.5

*Lethal time (LT_50_) values were calculated based on a generalized linear model (MASS package in R) from three replicates with 10 larvae per replicate. Confidence intervals (95%) for LT_50_ values are given in brackets. Marked values differed significantly from the LT_50_ value of the respective wild-type strain (p < 0.05)*.

† *significantly faster*;

* *significantly slower. If 50% mortality was not reached by the end of the experiment, LT_50_ values are given as >X, with X being the last time point at which the larvae were checked for survival. Sterile 0.9% NaCl served as control (Control). Each mutant was tested at least three times and results of at least two representative experiments are depicted. AprA, extracellular protease; Clp1391, cyclic lipopeptide of strain PCL1391; Fit, P. fluorescens insecticidal toxin; Gad, gluconate dehydrogenase; Gcd, glucose dehydrogenase; Hcn, hydrogen cyanide; Ofa, orfamide; Phl, 2,4-diacetylphloroglucinol; Phz, phenazine; Plt, pyoluteorin; Prn, pyrrolnitrin; Pvd, pyoverdine; Ses, sessilin*.

### *Plutella* feeding assays

Feeding assays were performed as described by Flury et al. (2016). Eggs of *P. xylostella* were obtained from Syngenta Crop Protection AG (Stein, Switzerland). General growth conditions for larvae before and during the experiments were 26°C, 60% humidity and a 16-h day, 8-h night cycle. One-week-old larvae (deriving from four different egg batches) were used for experiments during which each larva was kept separately in 128-cell bioassay trays (Frontier Agricultural Sciences, Delaware, USA) to prevent injuries due to cannibalism. The bottom of each well of the bioassay trays was covered by a wetted filter paper on which a pellet of insect diet was placed and inoculated with 10 μl of a suspension of washed bacterial cells. For insect diet preparation 500 ml of sterile ddH_2_O containing 7.5 g Agar was boiled for 1 min. Next, 50 g Adapta Bio-Dinkel (Hero Baby, Switzerland), one effervescent vitamin pill Santogen Gold (Coop, Switzerland), 15.5 g yeast extract, 7.5 g casamino acids, 0.25 g cholesterol, and 0.5 ml corn oil were added and all ingredients were mixed thoroughly with a blender. The hot mixture was poured into Petri dishes to a height of approximately 1.5 mm. From the solidified insect diet food pellets were cut with a sterile cork borer (4 mm diameter). For *P. protegens* CHA0 and its mutant derivatives, the experiments were performed with a bacterial dose of 4 × 10^6^ cfu/pellet and a total of 64 larvae per treatment. For *P. chlororaphis* PCL1391, *Pseudomonas* sp. CMR12a and derivatives, a dose of 2 × 10^7^ cfu/pellet was used and 32 larvae were tested for each treatment. Larvae were scored as live or dead regularly over time. Every mutant strain was tested at least in three independent experiments and two representative experiments are shown in Table 3. To exclude a human bias the experiments were performed double-blind as described above.

**Table 3:** **Lethal time 50 (LT_50_) for *Plutella xylostella* larvae upon oral uptake of wild-type and mutant *Pseudomonas* strains**.

**Strain**	**Phenotype**	**LT**_**50**_ **(h)**		
		**Experiment 1**	**Experiment 2**	**Experiment 3**
CHA0	Wild type	24.8 (23.7; 25.8)	23 (21.8; 24.1)	20.8 (19.8; 21.8)
CHA400	Pvd^−^	26.8 (25.3; 28.2)		23.6 (22.7; 24.6)^*^
CHA1196	Gcd^−^	21.9 (21.1; 22.7)^†^		23.6 (22.6; 24.6)^*^
CHA1197	Gad^−^	26.0 (24.9; 27.2)		23.0 (22.1; 24.0)^*^
CHA805-g	AprA^−^		22.1 (21.1; 23.2)	22.9 (21.8; 24.1)
CHA1241	Phl^−^	24.3 (23.3; 25.4)		22.9 (21.9; 23.8)^*^
CHA5091	Prn^−^	21.1 (20.0; 22.1)^†^		20.6 (19.6; 21.7)
CHA5092	Plt^−^	27.1 (25.8; 28.4)		21.7 (20.8; 22.6)
CHA5098	Phl^−^Prn^−^Plt	26.4 (25.2; 27.6)		19.7 (18.7; 20.8)
CHA5101	Ofa^−^	28.9 (27.7; 30.2)^*^		31.1 (29.3; 32.9)^*^
CHA5103	Hcn^−^	27.8 (26.4; 29.3)^*^		23.7 (22.8; 24.6)^*^
CHA5118	Phl^−^Prn^−^Plt^−^Hcn^−^Ofa^−^	31.6 (30.4; 32.7)^*^		34.6 (32.6; 36.5)^*^
CHA89	GacA^−^	>37	>37	>37
Control		>37	>37	>37
		**Experiment 1**	**Experiment 2**	
CMR12a	Wild type	18.0 (15.6; 20.5)	21.0 (20.0; 22.0)	
Clp1	Ses^−^	31.3 (28.0; 34.7)^*^	25.8 (23.8; 27.8)^*^	
ΔClp2	Ofa^−^	23.5 (21.2; 25.9)^*^	20.9 (19.7; 22.1)	
ΔClp2-clp1	Ofa^−^Ses^−^	37.3 (32.9; 41.7)^*^	33.5 (29.2; 37.9)^*^	
ΔPhz	Phz^−^	21.9 (20.4; 23.5)	20.8 (18.9; 22.7)	
ΔPhz-Clp1	Phz^−^Ses^−^	24.0 (22.3; 25.7)^*^	35.1 (31.5; 38.6)^*^	
ΔPhz-ΔClp2	Phz^−^Ofa^−^	20.1 (17.6; 22.6)	27.9 (26.3; 29.5)^*^	
ΔPhz-ΔClp2-Clp1	Phz^−^Ofa^−^Ses^−^	37.2 (33.5; 40.9)^*^	30.7 (27.7; 33.7)^*^	
Control		>42	>42	
		**Experiment 1**	**Experiment 2**	**Experiment 3**
PCL1391	Wild type	17.1 (15.7; 18.5)	20.9 (19.2; 22.5)	27.9 (25.4; 30.5)
PCL1832	Clp1391^−^	21.2 (19.4; 23.0)^*^	23.9 (21.5; 26.2)	
PCL1113	Phz^−^	19.5 (18.1; 20.9)	19.9 (18.1; 21.7)	
PCL1123	GacS^−^	37.6 (34.1; 41.2)^*^	34.0 (29.3; 38.8)^*^	
PCL5103	Hcn^−^		22.9 (21.7; 24.2)	30.9 (28.4; 33.4)
Control		>42	>42	>42

*Lethal time (LT_50_) values were calculated based on a generalized linear model (MASS package in R) from eight replicates with eight larvae per replicate in case of strain P. protegens CHA0 and from four replicates with eight larvae per replicate for strain P. chlororaphis PCL1391 and Pseudomonas sp. CMR12a. Confidence intervals (95%) for LT_50_ values are given in brackets. Marked values differed significantly from the LT_50_ value of the respective wild-type strain (p < 0.05)*.

† significantly faster;

* *significantly slower; If 50% mortality was not reached by the end of the experiment, LT_50_ values are given as >X, with X being the last time point at which the larvae were checked for survival. Sterile 0.9% NaCl served as control (Control). Each mutant was tested at least three times and results of two representative experiments are depicted. AprA, extracellular protease; Clp1391, cyclic lipopeptide of strain PCL1391; Gad, gluconate dehydrogenase; Gcd, glucose dehydrogenase; Hcn, hydrogen cyanide; Ofa, orfamide; Phl, 2,4-diacetylphloroglucinol; Phz, phenazine; Plt, pyoluteorin; Prn, pyrrolnitrin; Pvd, pyoverdine; Ses, sessilin*.

### Gene expression analysis

To study bacterial gene expression in the insect host, *G. mellonella* and *P. xylostella* were infected with *P. protegens* CHA0 as described above. For experiments with *G. mellonella*, gene expression was studied 20, 30, and 42 h post-injection. At 42 h infected larvae had died while control larvae were still alive. Three independent experiments were performed and each time a pool of 10 larvae was analyzed per time point. Hemolymph was collected from a cut near the tail of 10 surface disinfested larvae (20 s in 70% Ethanol, rinsed in sterile ddH_2_O), pooled (resulting in a total of 200–300 μl) and frozen in liquid nitrogen. *P. xylostella* larvae were collected after 20 h, 30 h, and as soon as death occurred. Three independent experiments were performed and each time a pool of 32 larvae was analyzed per time point. Larvae were washed in ddH_2_O, homogenized and frozen in liquid nitrogen. RNA was extracted from samples of both insect species using the Trizol-Reagent protocol™ (Thermo Fisher Scientific, Massachusetts, USA) and quantified with a NanoDrop™ 2000 (Thermo Fisher Scientific, Massachusetts, USA). To remove the remaining genomic DNA from the sample, a DNase treatment was performed with RNeasy mini Kit™ (Qiagen, California, USA). In order to verify the absence of contaminating genomic DNA, a PCR was performed with primers specific for the *16S rRNA* gene of *Pseudomonas* spp. (Bergmark et al., 2012). For each sample within one experiment, equal amounts of RNA were then converted into cDNA using the GoScript™ Reverse Transcription System (Promega, Wisconsin, USA) according to the instructions of the manufacturer. For each time point an equal amount of cDNA (6 ng in experiments one and three, 30 ng in experiment two) was amplified by PCR with Thermo Scientific™ DreamTaq™ DNA Polymerase (Thermo Fisher Scientific, Massachusetts, USA) using the primers listed in Supplementary Table S2. The presence of the amplicon was verified by electrophoresis in 1.5 or 3% agarose gels, depending on the expected size of the PCR product. The gel was post-stained with GelRed™ (Invitrogen, California, USA) and the bands visualized with the ChemiDoc™ XRS+ System (BioRad, California, USA).

### Statistics

Data analysis was performed in RStudio version 0.98.1017 (http://www.rstudio.com). To test for significant differences between survival curves of wild-type and mutant strains, the Log-Rank test of the Survival package of R was applied. LT_50_ values were calculated using a generalized linear model in the MASS package.

## Results

### Hydrogen cyanide mainly contributes to injectable insect toxicity

**Figure 1:**
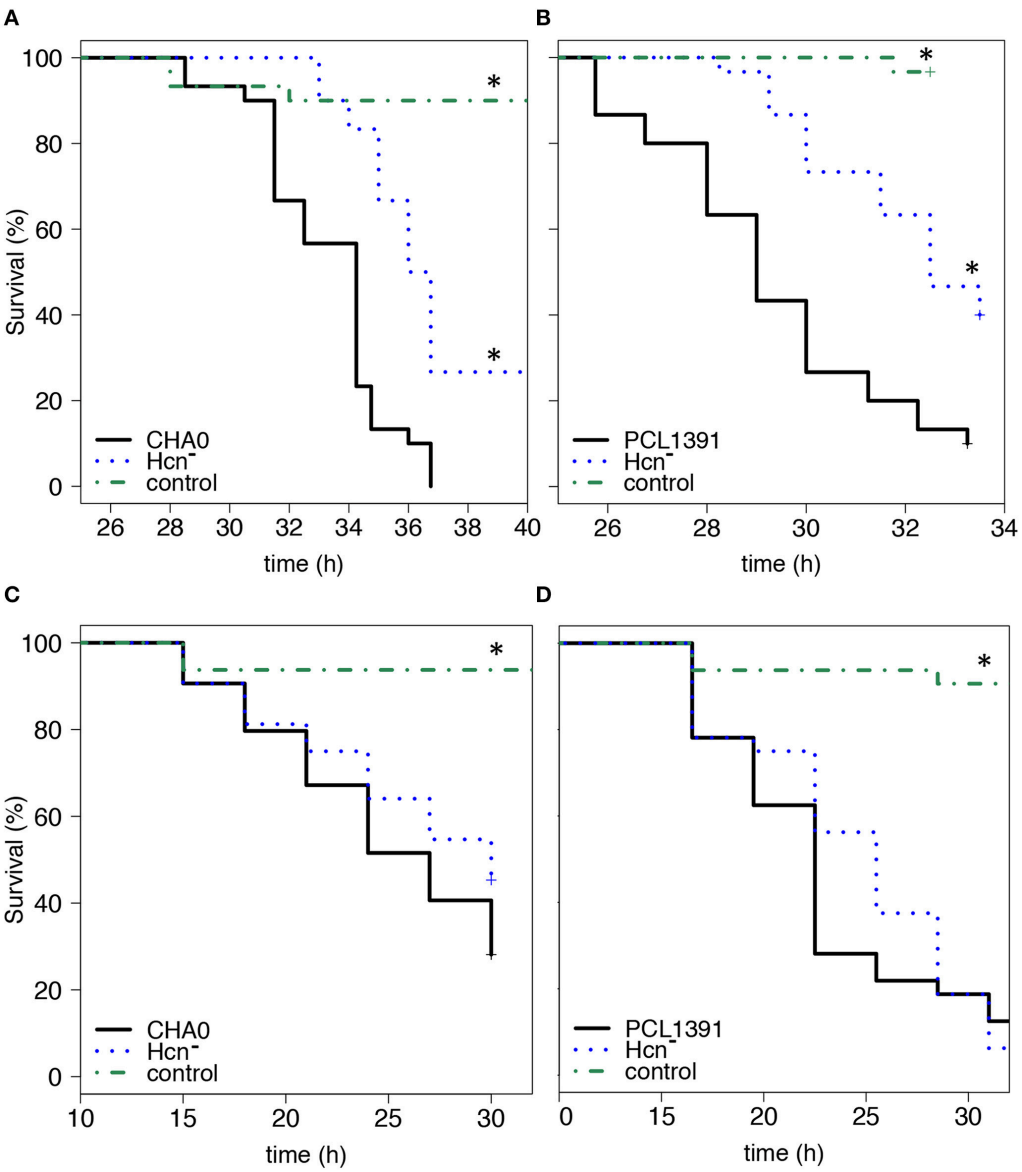
**Hydrogen cyanide-deficient mutants exhibit reduced injectable insecticidal activity. (A,B)** Injectable activity against *Galleria mellonella*. Thirty larvae per treatment were injected with 2 × 10^3^ bacterial cells and survival was recorded hourly. **(C,D)** Oral activity against *Plutella xylostella*. For *Pseudomonas protegens* CHA0 **(C)** 64 larvae were exposed to artificial diet inoculated with 4 × 10^6^ bacterial cells and for *Pseudomonas chlororaphis* PCL1391 **(D)** 32 larvae were exposed to 2 × 10^7^ bacterial cells. Sterile 0.9% NaCl solution served as control. Treatments that differed significantly from their respective wild-type strain (Log-Rank test *p* ≤ 0.05, Survival Package in R) are marked with an asterisk. This figure shows the survival curves of one representative experiment per strain and insect system. The LT_50_ values corresponding to these experiments and to a repetition of them are depicted in Tables 2, 3. Solid black line, wild-type strain; dotted blue line, mutants deficient for hydrogen cyanide production (Hcn^−^, CHA5103, PCL5103); dash-dot green line, 0.9% NaCl solution control.

In frame deletion mutants for hydrogen cyanide were generated for strains *P. protegens* CHA0 and *P. chlororaphis* PCL1391; however, several attempts to also create an Hcn^−^ mutant for strain CMR12a failed. The Hcn^−^ mutants of CHA0 and PCL1391 were not able to produce Hcn as confirmed with an Hcn-indicator paper assay (data not shown). When injected into the hemocoel of *G. mellonella* larvae, Hcn-deficient mutants of both strains killed the larvae significantly slower than the respective wild type according to a Log-Rank test (Figures 1A,B) and significantly higher LT_50_ values (Table 2). A similar trend was observed in feeding assays with *P. xylostella*. In two out of three experiments, the Hcn deficient mutant of CHA0 showed significantly higher LT_50_ values than the wild type (Table 3). However, the Log-Rank test between CHA0 and its Hcn-negative mutant CHA5103 was only nearly significant (*p* = 0.06) (Figure 1C) and for PCL1391 only a tendency for a longer kill-time, but no significant effects were found in oral infections (Figure 1D). Hence, the lack of Hcn seems to have a pronounced impact on injectable insecticidal activity, but rather a minor effect in oral infections.

### Different cyclic lipopeptides all contribute to insect toxicity

The impact of Clps on insecticidal activity was studied in all three *Pseudomonas* strains. Clp single and double mutants of CMR12a were already available (D'aes et al., 2011, 2014). Here, a mutant of CHA0 deficient for all three peptide synthases required for the production of orfamide A (Ma et al., 2016) was generated and termed CHA5101. This mutant neither reduced surface tension as indicated by a droplet collapse test nor showed swarming motility on soft agar plates (Supplementary Figures S1A,B). However, swarming ability was regained when plates were supplemented with orfamide A (Supplementary Figure S1A). Further, a Clp biosynthesis-defective mutant of PCL1391 with a Tn5 insertion in an *ofaC* homolog (PCL1832) was created and found to be impaired in causing droplet collapse (Supplementary Figure S1C). The Clp of PCL1391 will be called Clp1391 in the following. A detailed characterization of Clp1391 and the mutant PCL1832 will be presented elsewhere.

**Figure 2:**
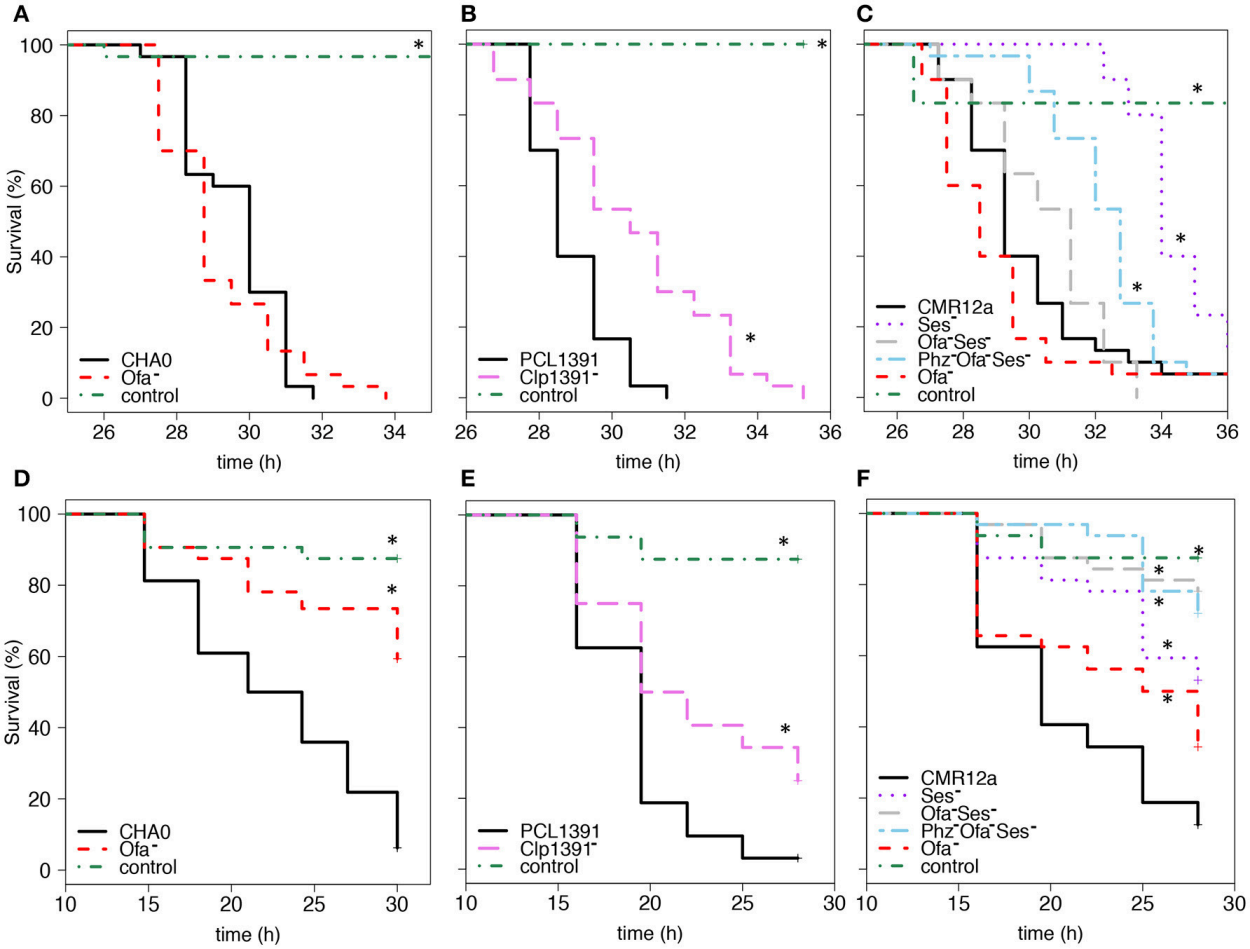
**Cyclic lipopeptides contribute to insecticidal activity of three plant-beneficial pseudomonads. (A,B,C)** Injectable activity against *Galleria mellonella*. 30 larvae per treatment were injected with 2 × 10^3^ bacterial cells and survival was recorded hourly. **(D,E,F)** Oral activity against *Plutella xylostella*. For *Pseudomonas protegens* CHA0 **(D)** 64 larvae were exposed to artificial diet inoculated with 4 × 10^6^ bacterial cells. In experiments with strains *Pseudomonas chlororaphis* PCL1391 **(E)** and *Pseudomonas* sp. CMR12a **(F)** 32 larvae were exposed to diet inoculated with 2 × 10^7^ bacterial cells. Sterile 0.9% NaCl solution served as control. Treatments that differed significantly from their respective wild-type strain (Log-Rank test *p* ≤ 0.05, Survival Package in R) are marked with an asterisk. This figure shows the survival curves of one representative experiment per strain and insect system. The LT_50_ values corresponding to these experiments and to a repetition of them are depicted in Tables 2, 3. Solid black line, wild-type strain; dashed red line, mutants deficient for orfamide production (Ofa^−^, CHA5101, CMR12a-ΔClp2), dashed pink line, mutant deficient for Clp1391 production (Clp1391^−^, PCL1832), dotted purple line, mutant deficient for sessilin production (Ses^−^, CMR12a-Clp1); dashed gray line, double mutant deficient for sessilin and orfamide production (Ses^−^Ofa^−^, CMR12a-ΔClp2-Clp1); dash-dot light blue line, triple mutant deficient for sessilin, orfamide and phenazine production (Ses^−^Ofa^−^Phz^−^, CMR12a-ΔPhz-ΔClp2-Clp1); dash-dot green line, 0.9% NaCl solution control.

When injected into the hemocoel of *G. mellonella* larvae the Ofa^−^ mutants of CHA0 and CMR12a did not kill slower than the wild-type strains, the Ofa^−^ mutant of CHA0 even killed significantly faster in one of the experiments (Figures 2A,C and Table 2). In contrast, the Clp1391^−^ mutant of PCL1391 killed always significantly slower than the wild parent strain, though, still causing 100% mortality at the end of the experiment (Figure 2B, Table 2). A clear reduction in virulence was also observed for the Ses^−^ mutant of CMR12a represented by significantly higher LT_50_ values in all repetitions of the experiment and also a lower killing rate (Figure 2C, Table 2).

In feeding experiments with *P. xylostella*, the Ofa^−^ mutant of CHA0 was always killing significantly slower than the wild type (Figure 2D, Table 3) and larval mortality after 30 h was at 40.7% compared to 96.9% for the wild type (Figure 2D). Similar observations were made for the Ofa^−^ mutant of CMR12a and the Clp^−^ mutant of PCL1391, although the effects were not always significant for these strains (Figures 2E,F; Table 3). Similar to the results for injectable insecticidal activity, Ses seems to make a major contribution to oral insecticidal activity of strain CMR12a. In all repetitions of the experiment, larval death caused by a Ses^−^ mutant was on average occurring at a later time point than death caused by the wild-type strain (Figure 2F, Table 3). Thus, after 28 h already 87.5% of larvae had died in the wild type treatment while mortality in the Ses^−^ mutant treatment was only at 46.9% (Figure 2F).

As Ses, Ofa, and Phzs are reported to influence each other's action (D'aes et al., 2014; Olorunleke et al., 2015), we also tested double and triple mutants of CMR12a. In *Galleria* injection experiments, a Ses^−^Ofa^−^ double mutant did not differ from the wild-type strain CMR12a, thus the Ses effect observed for the single mutant seems to disappear by the additional lack of Ofa. However, a triple mutant Phz^−^Ofa^−^Ses^−^, which in addition to not producing the two Clps neither produces Phz, performed similar to the Ses^−^ single mutant, killing *G. mellonella* larvae at a decreased pace compared to the wild type (Figure 2C, Table 2). In feeding experiments with larvae of *P. xylostella*, both the double mutant Ofa^−^Ses^−^ and the triple mutant Phz^−^Ofa^−^Ses^−^ caused higher LT_50_ values than the wild type (Table 3). Furthermore, Ses and Ofa seem to have additive effects in oral infections as the Ofa^−^Ses^−^ double mutant and the Phz^−^Ofa^−^Ses^−^ triple mutant showed not only significantly higher LT_50_ values compared to the Ofa^−^ single mutant, but also a strong and consistent tendency toward higher LT_50_ values compared to the Ses^−^ single mutant. Phz^−^Ses^−^ and Phz^−^Ofa^−^ double mutants did not kill more slowly than the respective Clp^−^ single mutants (Table 3).

### Mutants defective in the production of Phl, Prn, Plt and Phz are not impaired in insecticidal activity

*Pseudomonas* sp. CMR12a and *P. chlororaphis* PCL1391 both produce phenazine-1-carboxylic acid (PCA) and phenazine-1-carboxamide (PCN). Phzs are not only important for biocontrol of plant pathogenic fungi (Tambong and Höfte, 2001; Chin-A-Woeng et al., 2003; D'aes et al., 2011), but are further reported to be involved in biofilm formation and virulence (Mavrodi et al., 2006; Price-Whelan et al., 2006; Pierson and Pierson, 2010). However, in our experiments with Phz-deficient mutants of both strains, we did not find any reduction of virulence in infection assays with *G. mellonella* or *P. xylostella* larvae (Tables 2, 3, Supplementary Figure S2).

*P. protegens* CHA0 has been intensively studied for its production of Phl, Plt, and Prn, three metabolites contributing to antifungal activity of biocontrol pseudomonads on plant roots (Haas and Keel, 2003). We generated new in frame deletion mutants of CHA0 lacking biosynthesis genes needed for the production of these metabolites and screened them for insecticidal activity. All three mutants retained full toxicity in injection and feeding experiments (Tables 2, 3, Supplementary Figure S2). Because the production of different antifungal metabolites in *P. protegens* is tightly interlinked and the loss of one antibiotic might be compensated by the overproduction of another antibiotic (Haas and Keel, 2003; Baehler et al., 2005; Quecine et al., 2015; Clifford et al., 2016), we further created a triple mutant lacking the biosynthetic gene clusters for all three metabolites. In feeding experiments with *P. xylostella*, mortality caused by this mutant was not significantly different from that of the wild type (Table 3). However, a mutant that in addition to Phl, Prn, and Plt also lacks Ofa and Hcn production exhibited strongly reduced virulence compared to the wild type (Table 3). It further displays reduced virulence compared to single mutants defective for either Ofa or Hcn indicating an additive effect of these two mutations in the five-fold mutant. In contrast, the toxicity of the five-fold mutant did not differ from that of the wild type in injection experiments (Table 2). The fact, that not even a reduction in virulence due to the lack of Hcn production was observed, cannot be explained at present, but might be attributable to possible adverse effects on the production of other, yet to be discovered factors.

### No significant contribution of Pvd, AprA, Gcd and Gad to insect virulence

Finally, mutants of strain CHA0 deficient for the iron chelator Pvd, the two enzymes Gcd and Gad, which are involved in glucose utilization, or the AprA protease were tested in the systemic and oral insect infection assays, but none of them was found to behave differently from the wild type (Tables 2, 3). Although some of the strains were found to kill significantly faster or slower in one experiment, this result was not observed in other repetitions of the experiment. In some cases, even contradictory results were obtained in different repetitions, as for instance for the behavior of the Gcd^−^ mutant in *P. xylostella* experiments (Table 3).

### Gac-regulation

The GacA/GacS regulatory system is controlling a plethora of processes in fluorescent pseudomonads. *gac* mutants are deficient for the production of antifungal metabolites and generally strongly reduced in biocontrol against root pathogens (Lapouge et al., 2008; Hassan et al., 2010). Furthermore, it was shown that a *gacA* mutant is also reduced in oral insecticidal activity (Olcott et al., 2010; Ruffner et al., 2013; Flury et al., 2016; Loper et al., 2016), which was confirmed by our results. In oral infections of *P. xylostella*, a defect in the GacA/GacS regulatory system for strains *P. protegens* CHA0 and *P. chlororaphis* PCL1391 resulted in a drastic reduction of larval mortality and killing speed (Table 3, Supplementary Figure S2). Here, we tested Gac^−^ mutants of CHA0 and PCL1391 for the first time in injection experiments. In contrast to the feeding assays the effect of a *gacS* mutation in PCL1391 was very small in injection assays with *G. mellonella* larvae, i.e., resulting in a delay of killing of only about 1 h compared to the wild type (Table 2). Moreover, for GacA^−^ and GacS^−^ mutants of CHA0 not even a consistent significant effect was observed (Table 2). The fact that the Hcn^−^ mutant of CHA0 showed attenuated virulence toward *G. mellonella* larvae, but the Gac^−^ mutants did not, might be due to unknown factors, that are differentially expressed in Gac^−^ mutants compared to the wild type, compensating for the lack of Hcn production. In summary, the GacA/GacS regulatory system seems to be crucial for oral insecticidal activity of *P. chlororaphis* subgroup bacteria, but of limited importance when bacteria are directly injected into the hemocoel.

### Biosynthesis genes for antimicrobial compounds are expressed in insects

As a lack of neither Phl nor Prn or Plt affected virulence of *P. protegens* CHA0, we wondered whether these metabolites are actually produced in insects. We, therefore, checked the expression of the biosynthetic genes for these metabolites during infections upon injection of *G. mellonella* and during oral infections of *P. xylostella* by a qualitative PCR approach. Expression of *phlD, prnD*, and *pltA* was detected at early (20 h) and late time points (30 h) during systemic infections of *G. mellonella* larvae (Table 4, Supplementary Figure S3). Similarly, *prnD* was expressed at both time points during oral infections in at least two out of the three experiments (Table 4, Supplementary Figure S3). In contrast, expression of *phlD* was only observed in dead and never in infected, but still living *P. xylostella* larvae (Table 4, Supplementary Figure S3). Finally, expression of *pltA* during oral infection was only detected in experiment two in which higher amounts of cDNA were used than in the other experiments, indicating that expression levels of this gene during oral infection might be low.

**Table 4:** **Expression of biosynthetic genes for antifungal metabolites during insect infection by *Pseudomonas protegens* CHA0**.

	***16s***		***ofaA***		***phlD***		***fitD***		***prnD***		***hcnA***		***pltA***	
	**i**	**o**	**i**	**o**	**i**	**o**	**i**	**o**	**i**	**o**	**i**	**o**	**i**	**o**
20 h	3	3	0	0	3	0	3	1	2	2	3	1	3	1
30 h	3	3	3	2	2	0	3	3	2	3	3	2	3	1
Dead	3	3	3	3	3	3	3	3	3	3	3	3	3	3

*Galleria mellonella larvae were infected systemically by injection (i) of 2 × 10^3^ cells of P. protegens CHA0 into the hemocoel. Plutella xylostella larvae were infected orally (o) by feeding artificial diet inoculated with 4 × 10^6^ cells of P. protegens CHA0. For G. mellonella infections, hemocoel was collected 20 and 30 h post injection (living larvae) and after 42 h when larvae had succumbed to infection (Dead). For P. xylostella infections, entire larvae were collected after 20 and 30 h and when death occurred (Dead). Total RNA was extracted from insect tissue, converted into cDNA and gene expression for the indicated genes was checked by PCR. Because results for some of the genes varied, a summary of the outcome of three experiments is presented and numbers indicate how many times (out of three) expression of the respective gene was detected. A Pseudomonas specific primer for the 16s rRNA gene was used to detect presence of the bacteria. In non-infected control larvae, neither expression of the Pseudomonas 16s rRNA gene nor of any of the other genes was detected. For each gene and insect system one representative gel is shown in Supplementary Figure S3*.

We also tested the expression of genes required for the production of the insecticidal factors Ofa, Hcn, and FitD. In infections upon injection of *G. mellonella fitD* and *hcnA* were consistently expressed at both time points, while expression of *ofaA* was only detected after 30 h (Table 4). In oral infections all three genes were expressed in at least two out of tree experiments at the late time point. At time point 20 h, however, *ofaA* was never expressed and *hcnA* as well as *fitD* expression could only be detected in experiment two in which higher amounts of cDNA had been used (Table 4, Supplementary Figure S3).

## Discussion

**Figure 3:**
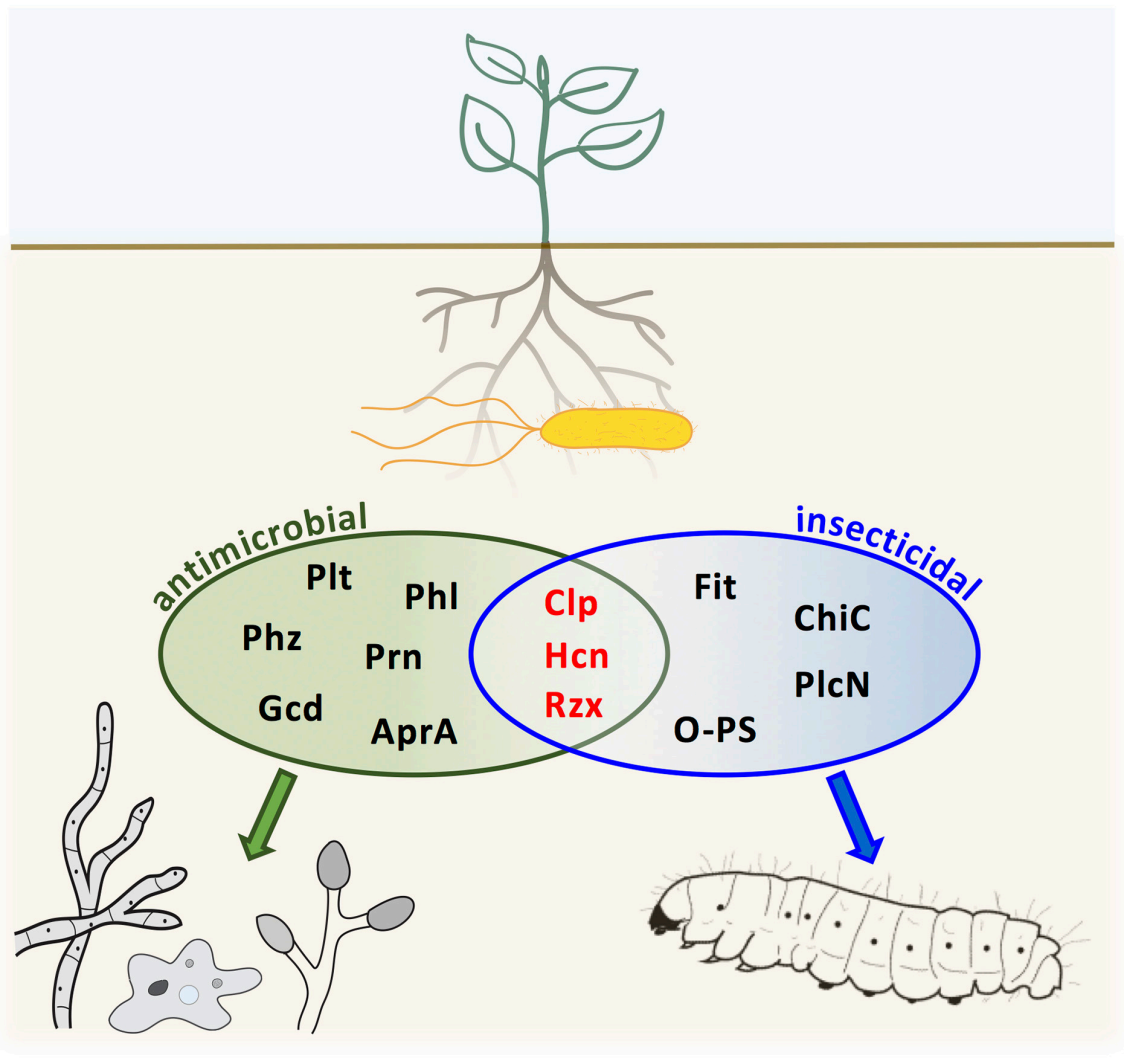
**Overview of exoproducts and cell surface components contributing to the antimicrobial and insecticidal activity of bacteria of the *Pseudomonas chlororaphis* subgroup**. Compounds involved in activity against both microorganisms as well as insects are depicted in red. AprA; protease AprA; ChiC, chitinase C; Clp, cyclic lipopeptide; Fit, *P. fluorescens* insecticidal toxin; Gcd, Glucose dehydrogenase; Hcn, hydrogen cyanide; O-PS, O-antigen polysaccharide; Phl, 2,4-diacetylphloroglucinol; Phz, phenazine; PlcN, phospholipase C; Plt, pyoluteorin; Prn, pyrrolnitrin; Rzx, rhizoxin.

This study presents an extensive mutational analysis with the aim to identify antimicrobial compounds that contribute to insecticidal activity of three different *Pseudomonas* biocontrol strains, *P. protegens* CHA0, *P. chlororaphis* PCL1391, and *Pseudomonas* sp. CMR12a. We identified Hcn as an important toxicity factor in strains CHA0 and PCL1391. In addition, mutants impaired in production of various Clps showed reduced insect toxicity in all three strains. Furthermore, we provide the first investigation on antibiotic production by a fluorescent pseudomonad in insect hosts. In summary, our results indicate that, indeed, certain antifungal compounds also contribute to anti-insect activity. To illustrate the knowledge on antimicrobial and insecticidal compounds produced by *P. chlororaphis* subgroup bacteria generated in this study and reported previously, we present an overview of major exoproducts and whether they are involved in activity against microbes or insects or both (Figure 3).

*P. protegens* CHA0 and *P. chlororaphis* PCL1391 lacking Hcn production showed a significant decrease of virulence when injected into the hemocoel of *G. mellonella* larvae. However, the effect was smaller and for PCL1391 there was only a tendency toward reduced toxicity when bacteria were ingested by *P. xylostella*. Cyanide is known to be toxic to most organisms as it inhibits the cytochrome-c oxidase, which is part of the mitochondrial respiratory chain (Way, 1984). Accordingly, cytochrome-c oxidase activity of termites was inhibited when they were exposed to Hcn produced by CHA0, finally causing insect death (Devi and Kothamasi, 2009). In injection experiments, cyanide was found to contribute to virulence of *Pseudomonas aeruginosa* PAO1 toward *D. melanogaster* (Broderick et al., 2008). In contrast, in a recent study by Loper et al. (2016) a lack of Hcn production in *P. protegens* Pf-5 did not impair its oral insecticidal activity against *D. melanogaster*. Both results are in line with our observation that a lack of Hcn production reduces toxicity of CHA0 and PCL1391 mainly in the hemolymph. We, thus, hypothesize that Hcn comes into play at a late stage of infection, once the bacteria already entered the hemolymph. Accordingly, at early stage of oral infections, when the bacteria assumingly still are in the gut, the Hcn biosynthesis gene *hcnA* of CHA0 was only expressed in one out of three experiments. However, strong expression of *hcnA* was observed in all experiments and at all stages when bacteria were injected into the larvae.

We tested mutants deficient for different Clps, i.e., orfamide A, orfamide B, Clp1391, and sessilin, and our results indicate that all of them contribute to insect toxicity exhibited by their producer strains. The strongest effect was found for sessilin, which is to our best knowledge the first report on the role of a tolaasin-like Clp in insect infection. The results obtained for *P. protegens* CHA0 are in line with former reports that orfamide A of strains F6 and Pf-5 contributes to toxicity against aphids and *D. melanogaster*, respectively (Jang et al., 2013; Loper et al., 2016). The main orfamide produced by CMR12a is orfamide B (Ma et al., 2016), and we provide first evidence that also this type of orfamide is contributing to insecticidal activity. The large size of the genes encoding the non-ribosomal peptide synthases responsible for the biosynthesis of Clps makes complementation of mutants extremely difficult. However, we believe that the detection of reduced toxicity by Clp^−^ mutants in three different strains, strongly suggests an important role for these molecules during insect infections. Still, how Clps contribute to insect infections, their target and mode of action remains speculative.

Clps are reported to be involved in many biological functions, such as motility, biofilm formation, protection against predators, and antagonism (Raaijmakers et al., 2010). On plants Clps produced by plant-beneficial bacteria were found to induce resistance and to contribute to plant protection against root pathogenic fungi (Raaijmakers et al., 2006; D'aes et al., 2010, 2011; Olorunleke et al., 2015). For instance the Clps massetolide A and viscosin of *P. fluorescens* SS101 and *P. fluorescens* SBW25, respectively, are able to cause lysis of zoospores of the oomycete pathogen *Phytophthora infestans*, the causal agent of tomato and potato late blight (De Souza et al., 2003; de Bruijn et al., 2007). In plant pathogenic *Pseudomonas*, Clps contribute to virulence by inserting into the host plasma membrane, forming pores, which leads to cell death and thus necrotic symptoms (D'aes et al., 2010). During infection, insecticidal pseudomonads do not remain in the gut, but cause fatal septicemia (Kupferschmied et al., 2013). Hence, upon oral uptake, bacteria have not only to survive the adverse conditions of the gut milieu and the insect immune response, but also to overcome the peritrophic membrane and the gut epithelial cells to gain access to the hemocoel (Vallet-Gely et al., 2008). Clps might be important in different ways during the infection of insects, on one hand for motility and biofilm formation during insect colonization and on the other hand to effectively damage the peritrophic membrane and/or insect blood cells, such as phagocytic hemocytes, involved in immunity against intruding pathogens (Lemaitre and Hoffmann, 2007). Recently, Sood et al. (2014) reported a role for Clps in insect infections. They identified paenilarvins, iturin-like Clps, in *Paenibacillus larvae*, a deadly bee pathogen causing American foulbrood. Paenilarvins, which also have activity against various filamentous fungi and yeasts, were found to cause significant mortality when fed to bee larvae (Sood et al., 2014; Müller et al., 2015). However, as for the Clps studied here, it is still elusive by which mechanisms paenilarvins affect insect larvae.

CMR12a is the only herein tested strain that produces two types of Clps, which is atypical for plant-beneficial pseudomonads. For plant-pathogenic *Pseudomonas* strains, however, which mostly produce one Clp each of the tolaasin and the syringomycin group (D'aes et al., 2010) it was suggested that the two Clps act synergistically, one as a toxin and the other as surfactant (Iacobellis et al., 1992; Batoko et al., 1998; Bender et al., 1999). Since our data imply that both Clps of CMR12a are involved in virulence toward insects, one could hypothesize a similar interplay of Ses and Ofa as described for Clps of *Pseudomonas syringae*. Motility and biofilm formation are often connected to bacterial pathogenicity. The Ofa^−^ mutants of CHA0 and CMR12a are impaired in swarming motility while the Ses^−^ mutant shows reduced biofilm formation (D'aes et al., 2014). In the Ofa^−^Ses^−^ double mutant of CMR12a the two mutations seem to partially compensate each other since the double mutant e.g. does produce more biofilm than the Ses^−^ single mutant (D'aes et al., 2014). This might explain our observation that the Ses^−^Ofa^−^ mutant is killing *G. mellonella* larvae more rapidly than the Ses^−^ single mutant. In general, a tight regulation between mobility and settlement seems to be crucial for full virulence of the herein studied bacteria against insects. While orfamides seem to be mainly important in oral infections of *P. xylostella*, a lack of Clp1391 and Ses also hampered infections of *G. mellonella* larvae upon injection. This may be due to different functions of structurally different Clps during the gut or the hemolymph phases of infection. However, it could be also attributed to a possible host specificity of certain toxicity factors, which cannot be excluded as we used two different model insects for injection and feeding experiments. For an in-depth analysis of the role of Hcn and Clps in insecticidal activity the mutants will have to be complemented. However, similar effects for mutants deficient in Hcn and Clp production could be observed for two (Hcn) and three (Clp) different *Pseudomonas* strains, which strongly indicates that these compounds contribute to insecticidal activity in these bacteria.

Besides Hcn and Clp, none of the other antimicrobial compounds investigated affected virulence in our test systems. The impact of phenazines on insecticidal activity of fluorescent pseudomonads was studied for the first time and the fact that Phz-deficient mutants of both CMR12a and PCL1391 did not differ in their toxicity from the respective wild type indicates that Phzs do not play a crucial role in insect infections by strains of the *P. chlororaphis* subgroup. Likewise, a pyocyanin deficient mutant of *P. aeruginosa* PAO1 was not reduced in virulence when injected into the hemocoel of the silkworm *Bombyx mori* (Chieda et al., 2008). Since Phzs are reported to contribute to *P. aeruginosa* virulence, e.g., during lung infections (Pierson and Pierson, 2010), Chieda et al. hypothesized that a possible pyocyanin effect might have been masked in their experiments as the investigated pyocyanin mutant was still able to produce other Phzs, such as PCA and PCN. However, the Phz^−^ mutants of PCL1391 and CMR12a do not produce any Phzs at all and still no reduction of virulence compared to the wild type could be observed.

Furthermore, Phl, Prn, and Plt show broad toxicity toward different organisms (Gross and Loper, 2009), but CHA0 mutants impaired in their production killed insects at the same pace as the wild type. This is in line with recent results by Loper et al. (2016) who neither found an effect of these antimicrobial compounds in oral toxicity of *P. protegens* Pf-5 toward *D. melanogaster*. However, we further studied the expression of antibiotic biosynthesis genes and our results imply that during infections by *P. protegens* CHA0 not only the biosynthetic genes for the insect toxicity factors Hcn and Ofa are expressed, but also the genes required for Phl, Prn, and Plt production. This indicates that these factors, although being dispensable for full virulence toward insects, still may have a function during insect colonization. As *prn* and *plt* biosynthetic genes are already expressed at early time-points in oral infections, when bacteria presumably are still colonizing the gut, it could be hypothesized that the respective compounds give an advantage in competition with other microbes present in the digestive tract of insects. This effect might thus be much more relevant in infections of natural insect populations harboring a more diverse microflora than the herein tested laboratory-grown animals. After larvae have succumbed to infection by *P. protegens* CHA0, the bacteria can use the cadaver as an optimal substrate for growth. At this stage, biosynthesis genes for all known antimicrobial compounds are expressed, which could again give a competitive advantage over other microbes trying to intrude the nutrient-rich insect substrate. Similarly, the entomopathogens *Photorhabdus* and *Xenorhabdus* produce numerous secondary metabolites to inhibit the growth of other microbes in the insect cadaver (Nielsen-Leroux et al., 2012).

The exoprotease AprA adds to the biocontrol potential of CHA0 against the root-knot nematode *Meloidogyne incognita* (Siddiqui et al., 2005). Further, AprA of the insect pathogen *Pseudomonas entomophila* is reported to contribute to virulence against *D. melanogaster* and was suggested to protect the bacteria against the insect immune response (Liehl et al., 2006). In the insect injection and feeding assays presented here, an *aprA* mutant of CHA0 was as virulent as the wild type. Similar results were obtained recently with an *aprA* mutant of Pf-5 (Loper et al., 2016) and an *aprX* mutant of CHA0 in oral infection assays (Flury et al., 2016). All these findings suggest that these exoproteases do not play an important role in insect infections by *P. protegens*. Similarly, a pyoverdine mutant was found to be as virulent as the wild type CHA0 confirming the results recently obtained with a *pvd* mutant of Pf-5 (Loper et al., 2016).

As demonstrated earlier (Olcott et al., 2010; Ruffner et al., 2013; Flury et al., 2016; Loper et al., 2016) Gac^−^ mutants were found to exhibit drastically reduced toxicity in oral insect infections. However, this was not the case in the injection experiments carried out in the present study. Hence, although factors positively controlled by the GacS/GacA regulatory system seem to be crucial for insect infection through the intestine, they seem to be irrelevant or compensated by other factors once the bacterium has entered the hemolymph.

In summary, the presented research enriches our knowledge on factors that are important for insecticidal activity of plant-beneficial pseudomonads. We were searching for compounds contributing to dual activity against microbes and insects and discovered that in fact Hcn and Clps exhibit such versatile functions (Figure 3). Our study has unraveled the cyclic lipopeptides orfamides A and B, Clp1391 and sessilin as further strain-specific insect virulence factors besides rhizoxin (Loper et al., 2016) and O-antigen polysaccharides (Kupferschmied et al., 2016) in plant-beneficial pseudomonads. Hcn can be added to the anti-insect arsenal common to all strains of the *P. chlororaphis* subgroup, i.e. Fit toxin, chitinase C, and phospholipase C (Flury et al., 2016). Nevertheless, insecticidal activity seems to be orchestrated by various factors and many of them are presumably still awaiting discovery until a concluding picture on insect virulence in the *P. chlororaphis* subgroup can be drawn (Keel, 2016). Moreover, how the herein described toxicity factors intervene during insect infection, their targets and modes of action warrant further investigation. Besides the identification of new toxicity factors our study is the first to investigate expression of biosynthesis genes of antibiotic compounds during insect infection and our results indicate that antifungal metabolites may not only be of importance on plant roots, but presumably also play some role in insect hosts. This points to an even larger versatility of these compounds than assumed so far. In general, *P. protegens* CHA0, *P. chlororaphis* PCL1391 and *Pseudomonas* sp. CMR12a seem to possess an arsenal of weapons to compete in different habitats ranging from roots to insects and maybe even other yet to be discovered niches. It will be a fascinating task to further explore, how they switch between their different life-styles as plant-colonizing beneficial bacteria and insect-colonizing pathogenic bacteria.

## Author contributions

PF, SW, GB, CK, and MM designed the research; GB, MH, CK, and MM supervised the study; PF, MP, PK, KK, ZoM, and SW generated and characterized the mutants; PF, NA, NH, SS, PV, and ZaM performed the insect experiments; PV and FD performed the gene expression analysis; PF analyzed the data and wrote the paper together with SW, GB, MH, CK, and MM. All authors critically revised the manuscript and approved the final version.

## Funding

The study was funded by grants of the Swiss National Foundation for Scientific Research SNSF (Projects 31003A-138248, 31003A-159520, NRP68 406840_143141 and 406840_161904). KK was supported by a Scholarships for Foreign Students of the Swiss Government. PF was supported with a “Mobility Grant for Research Stays” granted by the Swiss Plant Science Web. ZoM (Ghent University) was supported by scholarships from China Scholarship Council (CSC, No. 201204910376) and a special research fund (Bijzonder Onderzoeksfonds, BOF) from Ghent University.

### Conflict of interest statement

The authors declare that the research was conducted in the absence of any commercial or financial relationships that could be construed as a potential conflict of interest.
