# Supplementary material for: Antimicrobial and Insecticidal: Cyclic Lipopeptides and Hydrogen Cyanide Produced by Plant-Beneficial Pseudomonas Strains CHA0, CMR12a, and PCL1391 Contribute to Insect Killing
Source: Front Microbiol. 2017 Feb 3;8:100. doi: 10.3389/fmicb.2017.00100 (PMC5289993; doi:10.3389/fmicb.2017.00100)
Supplement: Supplementary file 1 [file DataSheet5.pdf]

Supplementary Material

Antimicrobial and insecticidal: cyclic lipopeptides and hydrogen cyanide produced by plant-beneficial *Pseudomonas* strains CHA0, CMR12a and PCL1391 contribute to insect killing

Pascale Flury[1], Pilar Vesga[1], Maria Péchy-Tarr[2], Nora Aellen[1], Francesca Dennert[1], Nicolas Hofer[1], Karent Paola Kupferschmied[2], Peter Kupferschmied[2], Zane Metla[1,3], Zongwang Ma[4], Sandra Siegfried[1], Sandra de Weert[5, 6], Guido Bloemberg[5,7], Monica Höfte[4], Christoph Keel[2]* and Monika Maurhofer[1]*

*** Correspondence:** Monika Maurhofer: monika.maurhofer@usys.ethz.ch and Christoph Keel: christoph.keel@unil.ch

Content

Supplementary Table S1. Plasmids and primers used to create deletion mutants.

Supplementary Table S2. Primers used for qualitative gene expression analysis.

Supplementary Figure S1. Characterization of Clp mutants of *P. protegens* CHA0 and *P. chlororaphis* PCL1391.

Supplementary Figure S2. Antifungal metabolites without significant impact on oral insecticidal activity against *Plutella xylostella*.

Supplementary Figure S3. Expression of biosynthetic genes for antifungal metabolites of *Pseudomonas protegens* CHA0 during insect infections.

References

Supplementary Table S1. Plasmids and primers used to create deletion mutants

Plasmid or oligonucleotide	Relevant characteristics or sequence (5′ →3′)	Reference or comment
Plasmids		
pEMG	pSEVA212S; *ori*R6K, *lacZα* MCS flanked by two I-SceI sites; Km^{r}, Ap^{r}	Martinez-Garcia and de Lorenzo (2011)
pSW-2	*ori*RK2, *xylS*, P_m::*I-sce*I; Gm^{r}	Martinez-Garcia and de Lorenzo (2011)
pME8314	pEMG-Δ*prnABCD*; suicide plasmid for the deletion of the *prnABCD* gene cluster (PFLCHA0_c36450 to PFLCHA0_c36480) in CHA0; Km^{r}	This study
pME8315	pEMG-Δ*pltABCDEFG* suicide plasmid for the deletion of the *pltABCDEFG* gene cluster (PFLCHA0_c28450 to PFLCHA0_c28510) in CHA0; Km^{r}	This study
pME8322	pEMG-Δ*hcnABC* suicide plasmid for the deletion of the *hcnABC* gene cluster (PFLCHA0_c26420 to PFLCHA0_c26440) in CHA0; Km^{r}	This study
pME8325	pEMG-Δ*ofaABC*; suicide plasmid for the deletion of the *ofaABC* gene cluster (PFLCHA0_c21860 to PFLCHA0_c21880) in CHA0; Km^{r}	This study
pME8400	pEMG-Δ*phlACBD* suicide plasmid for the deletion of the *phlACBD* gene cluster (PFLCHA0_c59080 to PFLCHA0_c59110) in CHA0; Km^{r}	This study
pME11047	pEMG-Δ*hcnABC* suicide plasmid for the deletion of the *hcnABC* gene cluster (PCL1391_2240 to PCL1391_2242) in PCL1391; Km^{r}	This study
Primers		
ofaABC-1	CGGAATTCCCGATGAACCTGATCCAGTTCT, EcoRI	Deletion of CHA0 *ofaABC*
ofaABC-2	GGGGTACCCAGTTGGTCGAGCCAGATATC, KpnI	Deletion of CHA0 *ofaABC*
ofaABC-3	GGGGTACCAACCACTTCAGCCTGCTCAAGG, KpnI	Deletion of CHA0 *ofaABC*
ofaABC-4	CGGGATCCAGTCACGGTAGCGCTCGTAGAT, BamHI	Deletion of CHA0 *ofaABC*
prn-1	GGAATTCAATTGGCTCAAGGACAGTTGGTTC, EcoRI	Deletion of CHA0 *prnABCD*
prn-2	CCCAAGCTTCATAACACTCCCTGTTTCGAGG , HindIII	Deletion of CHA0 *prnABCD*
prn-3	CCCAAGCTTAAGTACCGTGCGTTCTACCGC, HindIII	Deletion of CHA0 *prnABCD*
prn-4	CGGGATCCGCGAGCGTATCTTTCGAGACG, BamHI	Deletion of CHA0 *prnABCD*
PphlACBD-1	GGGGTACCCCTTAAGAGATTAGATCGTCTG, KpnI	Deletion of CHA0 *phlACBD*
PphlACBD-2	GGAATTCGTCATAGGGATTGGTGCAGGTGC, EcoRI	Deletion of CHA0 *phlACBD*
PphlACBD-3	GGAATTCAACCTCAATCGCGGCGACATCGG, EcoRI	Deletion of CHA0 *phlACBD*
PphlACBD-4	GCTCTAGAGACAATGATGCTGGTGGGGGTG, XbaI	Deletion of CHA0 *phlACBD*
plt-1	GGAATTCAGGTGGGATGCCAAGTAGTCT, EcoRI	Deletion of CHA0 *pltABCDEFG*
plt-2	CCCAAGCTTCATAGACGTACGCTCCTGCA, HindIII	Deletion of CHA0 *pltABCDEFG*
plt-3	CCCAAGCTTGTGTGAGCCGACTATTGGGC, HindIII	Deletion of CHA0 *pltABCDEFG*
plt-4	CGGGATCCGACGGCGAACACACTAAAATCC, BamHI	Deletion of CHA0 *pltABCDEFG*
hcn-1	CGGAATTCAGGCCGTGGAAGAAGCCAAGCA, EcorI	Deletion of CHA0 *hcnABC*
hcn-2	GGGGTACCTATCTGACGCATTGTGGGTTCA, KpnI	Deletion of CHA0 *hcnABC*
hcn-3	GGGGTACCGTGAAGAAACAGCCCGAACCGG, KpnI	Deletion of CHA0 *hcnABC*
hcn-4	CGGGATCCTTTCGGTGTTCAGCACCTTCGA, BamHI	Deletion of CHA0 *hcnABC*
hcn1391-del-1	GGAATTCAAGTCATCGCCAGCCTCGAGGCC, EcoRI	Deletion of PCL1391 *hcnABC*
hcn1391-del-2	CCCAAGCTTTTCGGTATGGCGCATCAGGAA, HindIII	Deletion of PCL1391 *hcnABC*
hcn1391-del-3	CCCAAGCTTTTGCAGGTGGCGTTGGCCTGA, HindIII	Deletion of PCL1391 *hcnABC*
hcn1391-del-4	CGGGATCCGACGACACGGTGCTGATGGATT, BamHI	Deletion of PCL1391 *hcnABC*

Ap^{r}, ampicillin; Gm^{r}, gentamicin; and Km^{r}, kanamycin resistance, respectively.
Specified restriction sites are underlined.

Supplementary Table S2. Primers used for qualitative gene expression analysis.

Target gene	Primers	Sequence (5'→3')	Annealing temp.	Reference or comment
phlD	PhlD_65F_DEG<br>PhlD_236R_DEG	GGT RTG GAA GAT GAA RAA RTC<br>GCC YRA BAG YGA GCA YTA C	50°C<br>(55°C)	This study
fitD	FitD_66F_DEG<br>FitD_308R_DEG	CTA TCG GGT SCA GTT CAT CA<br>TTC TTG TCG GSA AAC CAC T	60°C	This study
prnD	PrnD_F<br>PrnD_R	TGC ACT TCG CGT TCG AGA C<br>GTT GCG CGT CGT AGA AGT TCT	60°C	Garbeva et al. (2004)
Pseudomonas spp., 16s rRNA	Pse435F<br>Pse686R	ACT TTA AGT TGG GAG GAA GGG<br>ACA CAG GAA ATT CCA CCA CCC	60°C	Bergmark et al. (2012)
hcnA	HcnA_F<br>HcnA_R	CGG GCT CAA GTT CGT CAT CT<br>AAG TAC ACA TCC ACG CCG TT	60°C	This study
pltA	PltA_F<br>PltA_R	TGA CGT CGA GTT TCT CAG CC<br>GGT CAT CGG CAG GAA GTG AA	60°C	This study
ofaA	OfaA_F<br>OfaA_R	GGC CTG CTC TAT CAC CAC ATG<br>CCT GCC ATT CTT GAA CCG TCA	60°C	This study

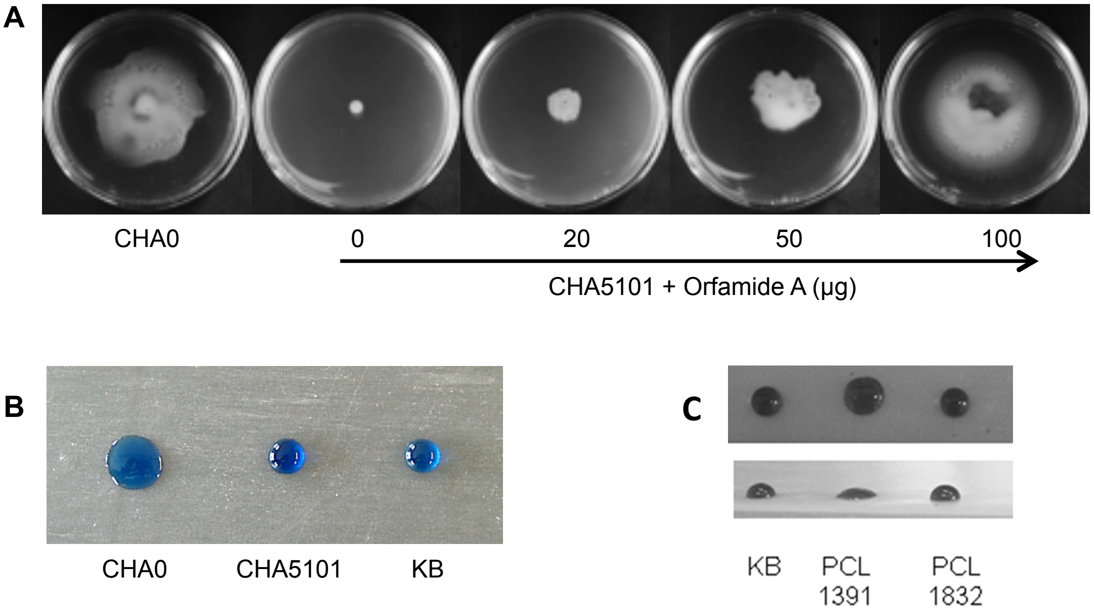

Supplementary Figure S1. Characterization of Clp mutants of *Pseudomonas protegens* CHA0 and *Pseudomonas chlororaphis* PCL1391. (A) Surface swarming motility of *P. protegens* CHA0 and its isogenic Δ*ofaABC* mutant CHA5101. 5 μL of washed bacterial cells were dropped to the center of soft agar LB plates (0.6% w/v agar). Pictures were taken after 20 h of incubation at 28 °C. The orfamide mutant CHA5101 was complemented by adding orfamide A of *P. protegens* CHA0 to the medium at the indicated concentrations (μg/plate). **(B, C)** Droplet collapse assay. Supernatants of KB overnight cultures of *P. protegens* CHA0, *P. chlororaphis* PCL1391 and their isogenic mutants CHA5101 und PCL1832 (insertional mutation in *ofaC* homolog) were spotted on parafilm and observed for loss of surface tension. Evan blue solution **(B)** or methylene blue **(C)** were added for photographic purposes and had no influence on the shape of the droplets. Experiments were repeated independently for three times with similar results and representative pictures are shown.

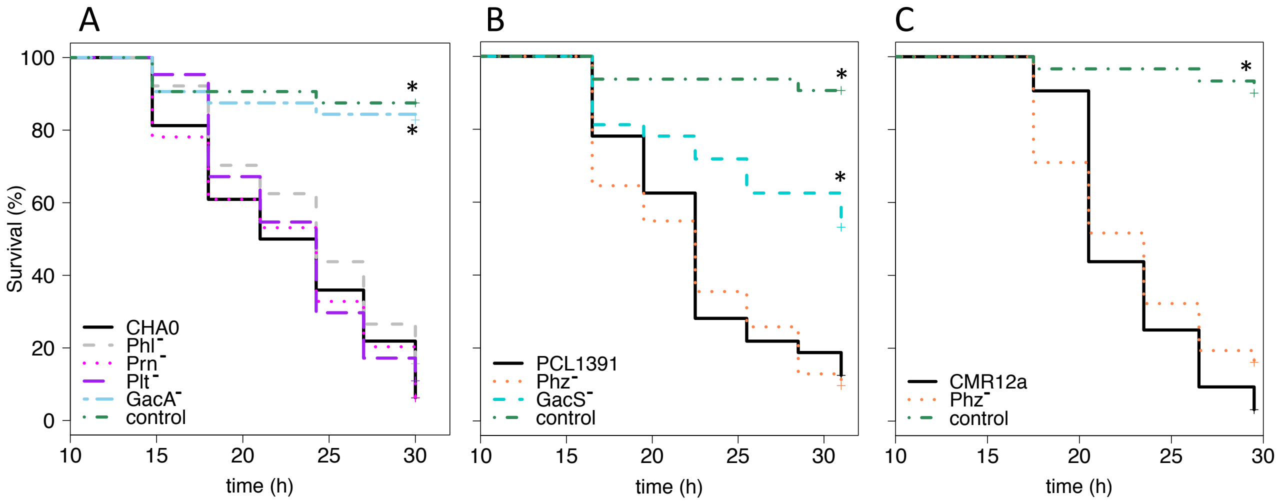

Supplementary Figure S2. Antimicrobial metabolites without significant impact on oral insecticidal activity of selected pseudomonads against *Plutella xylostella*. For *Pseudomonas protegens* strain CHA0 **(A)** 64 larvae were exposed to artificial diet inoculated with 4 x 10^6 bacterial cells. In experiments with strain *Pseudomonas chlororaphis* strain PCL1391 **(B)** and *Pseudomonas* sp. CMR12a **(C)** 32 larvae were exposed to 2 x 10^7 bacterial cells. Treatments that differed significantly from their respective wild-type strain (Log-Rank test $p \leq 0.05$, Survival Package in R) are marked with an asterisk. LT_{50} values of the experiments presented above and of a repetition of the experiment are listed in Tables 2 and 3. Each strain was tested at least three times and similar results were obtained. Solid black line, wild-type strain; dashed grey line, mutant deficient for 2,4-diacetylphloroglucinol production (Phl^-, CHA1241); dotted pink line, mutant deficient for pyrrolnitrin production (Prn^-, CHA5091); dashed purple line, mutant deficient for pyoluteorin production (Plt^-, CHA5092); dash-dot light blue line, mutant deficient for GacA production ($GacA^-$, CHA89); orange dotted lines, mutants deficient for the production of phenazines (Phz^-, PCL1113, CMR12a-ΔPhz); dashed turquois line, mutant deficient for GacS production ($GacS^-$, PCL1123); dash-dot green line, 0.9% NaCl solution control.

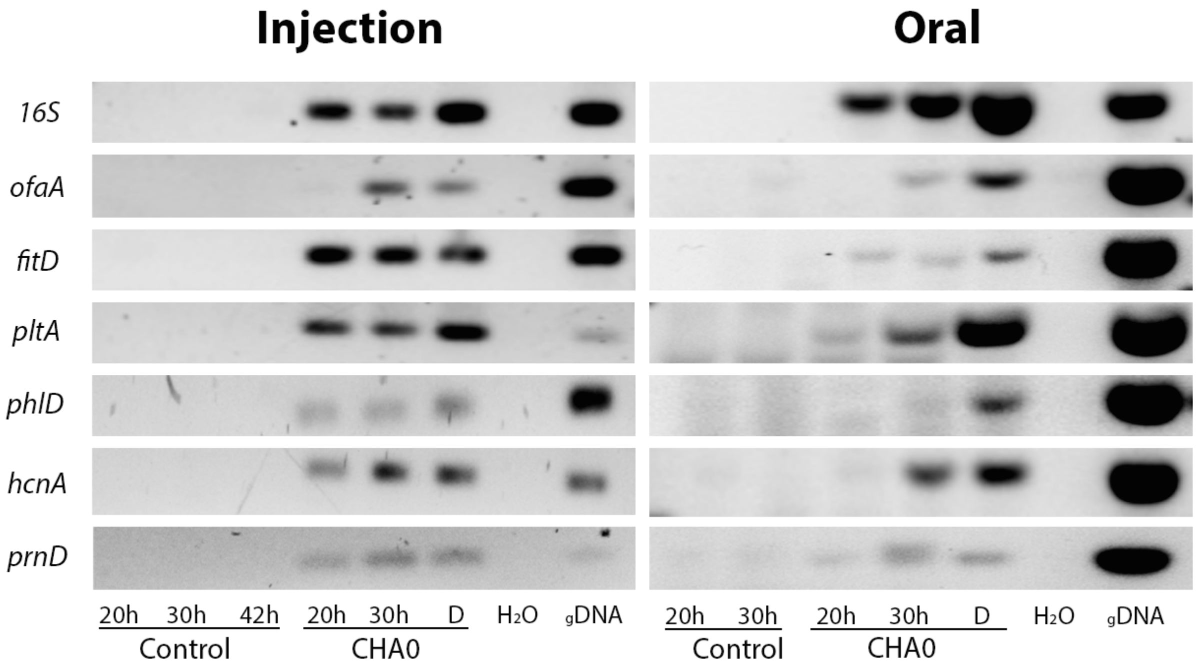

Supplementary Figure S3. Expression of biosynthetic genes for antimicrobial metabolites of *Pseudomonas protegens* CHA0 during insect infections. *Galleria mellonella* larvae were infected systemically by injection of 2 x 10^3 cells of *P. protegens* CHA0 (Injection). Hemocoel was collected 20 h and 30 h post injection (living larvae) and after 42 h when larvae had succumbed to infection. *Plutella xylostella* larvae were infected orally by feeding artificial diet inoculated with 4 x 10^6 cfu (Oral). Larvae were collected after 20 h, 30 h, and when death occurred (D). Total RNA was extracted from hemolymph (*Galleria*) or entire insects (*Plutella* larvae), converted into cDNA, and gene expression for the indicated genes was determined by PCR using specific primers (Supplementary Table S2). Control larvae were treated with sterile 0.9% NaCl solution. Genomic DNA of CHA0 (gDNA) served as positive control. The experiment was performed three times. For each gene and insect system one representative gel is shown.

References

Bergmark, L., Poulsen, P.H., Al-Soud, W.A., Norman, A., Hansen, L.H., and Sorensen, S.J. (2012). Assessment of the specificity of *Burkholderia* and *Pseudomonas* qPCR assays for detection of these genera in soil using 454 pyrosequencing. *FEMS Microbiol Lett* 333, 77-84.

Garbeva, P., Voesenek, K., and Van Elsas, J.D. (2004). Quantitative detection and diversity of the pyrrolnitrin biosynthetic locus in soil under different treatments. *Soil Biology & Biochemistry* 36, 1453-1463.

Martinez-Garcia, E., and De Lorenzo, V. (2011). Engineering multiple genomic deletions in Gram-negative bacteria: analysis of the multi-resistant antibiotic profile of *Pseudomonas putida* KT2440. *Environ Microbiol* 13, 2702-2716.
